# Prebiotic plausibility and networks of paradox-resolving independent models

**DOI:** 10.1038/s41467-018-07274-y

**Published:** 2018-12-12

**Authors:** Steven A. Benner

**Affiliations:** Foundation for Applied Molecular Evolution, Westheimer Institute for Science and Technology, 13709 Progress Blvd., Alachua, FL 32615 USA

## Abstract

The plausibility of any model in science comes from the extent of its interconnections to other models that are grounded in different premises and reasoning. Focusing research on paradoxes in those models, logic whereby they appear to generate unacceptable conclusions from seemingly indisputable premises, helps find those interconnections.

It is difficult to identify the very first use of the word "plausibility" as a concept to constrain models that describe the emergence of life on Earth as a historical event. Nevertheless, the concept was sufficiently prevalent by 1974 for Orgel and Lohrman to use the word “plausible” a half-dozen times in their classic review of prebiotic chemistry^1^. That review referenced the term to earlier articles in *Nature*^2^ and *Science*^3^.

Specifically, Orgel and Lohrmann argued that a chemical reaction sequence involving precursor molecules was "plausible" if those precursors could be seen in interstellar dust clouds. While their logic was not spelled out, Orgel and Lohrmann likely considered two lines of reasoning. A demanding line of reasoning would hold, as a premise, that molecules in planet-forming dust clouds remain intact during planetary accretion and thus remain available for the origins of life. A separate line of reasoning does not require this premise, but rather uses the presence of these molecules in dust clouds as evidence for the existence of abiological processes for their synthesis, processes that may have produced them independently on a post-accretion Earth.

The second line of reasoning is today considered more defensible, given that the Earth suffered very high temperatures during its accretion, temperatures that would leave few organic molecules intact. Thus, the presence of adenine in carbonaceous chondrites is taken as evidence that adenine was available prebiotically, not because meteoritic adenine arrived in quantities sufficient to support the prebiotic synthesis of RNA, but because it indicates the existence of abiological processes that might have made adenine on post-accretion Earth, just as they evidently did on the parent body of chondrites.

It was 30 years before Orgel confronted the easy transformation of “prebiotically plausible” from a phrase supported by examinable premises to instead mean: “A molecule that I desire for my model”^4^. In 2004, Orgel offered three criteria to adjudicate the prebiotic plausibility of individual molecules. The first was circular (its precursors must be prebiotically plausible). The second (reactions forming the molecule must occur in water) was rich in assumptions that excluded alternative solvents^5^. The third was subjective (the yield of molecule must be “significant”).

Orgel concluded by suggesting that “it would not be wise to define too closely” the concept of “prebiotically plausible”. Aside from being another example of the endorsement of semantic and philosophical imprecision by heroes in this field^6^, this does not offer the editors of journals guidance when evaluating manuscripts that purport to present investigator-managed chemistry, much done in Pyrex^7^, as relevant to origins.

This brings us to today. A half-century after the widespread use of the concept, a *Nature Communications* editor has solicited comments to put substance behind the phrase.

A single example illustrates the challenge. Hydrogen cyanide (HCN) is on nearly every list of prebiotically plausible precursors for biomolecules. It is seen in planet-forming dust clouds. It is observed in the atmospheres of gas giant planets in our Solar System. It was made by Stanley Miller by electrical discharge through mixtures of methane (CH_4_), ammonia (NH_3_), and water (H_2_O). These are touchstones of “prebiotic plausibility”. HCN, as well as cyanamide (H_2_NCN), cyanogen (NCCN), and cyanoacetylene (HCCCN), are easy precursors of the nucleobases seen in RNA (including adenine), allowing these molecules to also appear on these lists.

Unfortunately, current theory holds that Earth’s native atmosphere was more oxidizing than the Miller atmosphere. Carbon was more likely present as carbon dioxide (CO_2_), not methane. Nitrogen was more likely present as dinitrogen (N_2_), not ammonia. This model is supported by detailed studies of rocks surviving from that time^8^. More unfortunately, such atmospheres are very bad sources of HCN, HCCCN, and the other reduced molecules on these lists of prebiotically plausible compounds, including those in popular models for the prebiotic synthesis of adenine. Thus, the prebiotic plausibility of HCN, the other molecules, and adenine long ago vanished as Earth-made species, even though literature too voluminous to cite here continues to assume otherwise.

This creates a paradox. If one premises that life originated via an RNA-First prebiotic process that used adenine as a precursor and that adenine was formed from HCN from a Hadean terran atmosphere, then the premises that view HCN as an impossible product of our early atmosphere force the conclusion that life could not have originated on Earth. An unacceptable conclusion follows by the force of logic from seemingly acceptable premises.

Such paradoxes are useful^9^ because they narrow the scope of research, identifying premised assumptions that must be examined in greater detail. For example, researchers might re-examine the premise that assumes in situ formation of adenine. This would force them to reconsider how much adenine could possibly have arrived by meteorite to accumulate in “warm little ponds”^10^.

Alternatively, the paradox might force researchers to re-examine the premise that adenine is required to form adenosine, the combination of adenine and ribose. They then must develop prebiotic routes to RNA that do not join ribose directly to pre-formed adenine^11^. Such focus is important in this field, as many parameters are unconstrained because of the antiquity of the origins event; we cannot study any more than a few.

A paradox is quite different from a "difficult problem" precisely because it generates an unacceptable conclusion (“Life could not have originated…”) by logic from seemingly established premises. A “difficult problem” has no such logical framework. As an example, Inoue et al. showed by experiment 35 years ago that RNA could be formed by condensation of nucleotide imidazolides on a template^12^. How this actually works is an archetypal difficult problem; it took 35 years to solve^13^. However, no logic reasoned from established premises that life could not have originated without this reaction, or that we must understand its mechanism to understand its role in the origins of life.

Their ability to enforce focus in an otherwise unfocused field allows paradoxes to orient research programs. For example, Genda, Brasser, and Mojzsis recently revised the model for Earth’s Hadean atmosphere as part of a paradox-focused research program managed by the Foundation for Applied Molecular Evolution, supported by the John Templeton Foundation^14^. Here, they re-examined data related to the late veneer of the Earth and Moon.

Analysis of the late veneer starts with a generally accepted premise that the reducing power of early Earth was quickly lost early by the gravitational sinking of heavy metallic iron to its core. This is attested by the aforementioned high oxidation state of the early Earth mantle.

However, the iron as it sank should have taken with it iron-loving siderophilic elements, such as manganese, cobalt, nickel, platinum, and gold. The presence of these elements in today’s crust is evidence for their delivery as a late veneer in subsequent accretion events, events too small to re-melt the planet to allow their siderophiles to also sink to Earth’s core. The questions then become: How many subsequent accretion events delivered the late veneer? Many small events, which would have no impact on the redox state of the atmosphere? Or a few bigger events by impactors that themselves had iron cores? These, disrupted by the impact, would have delivered reducing iron metal to the Hadean atmosphere.

Inspecting the relative amounts of veneer on the Moon and the Earth, Genda et al. found data to support the second model. Chemists in the paradox-focused program understood the significance of their model for adenine. Iron delivered to the oxidized Hadean atmosphere containing CO_2_, N_2_, and H_2_O would put it out-of-redox-equilibrium with the crust, perhaps for 100 million years. This iron-reduced atmosphere would be a productive generator of HCN, HCCCN, NCCN, and other reduced organic molecules; these would be delivered to the oxidized crust. This would allow reduced organics to be in contact with the oxidized mineral species sometimes invoked in prebiotic RNA synthesis (borate, molybdate, sulfur dioxide)^7^. HCN would then be back, with HCCCN, NCCN, and other reduced organic molecules now all prebiotically plausible.

However, the argument for their plausibility is no longer a half-argument with gaps in the reasoning and a large amount of “I wish” bias. Instead, it is contingent on a model for planetary formation, one entirely independent of any Pyrex chemistry, its supporting theory, or  a chemist's desires. The planetary model may be evaluated under its own standards-of-proof^15^, and will rise or fall based on criteria quite independent of criteria that are used to evaluate chemical models. Nothing is ever proven in science. However, a network of models, each subject to independent test in their own fields, makes the big picture more, shall we say, plausible.
